# Proteomics reveals that cell density could affect the efficacy of drug treatment

**DOI:** 10.1016/j.bbrep.2022.101403

**Published:** 2022-12-09

**Authors:** Zhichao Xue, Jiaming Zeng, Yongshu Li, Bo Meng, Xiaoyun Gong, Yang Zhao, Xinhua Dai

**Affiliations:** a Technology Innovation Center of Mass Spectrometry for State Market Regulation, Center for Advanced Measurement Science, National Institute of Metrology, Beijing, 100029, PR China; b Shenyang University of Chemical Technology, College of Chemical Engineering, Shenyang, 110142, PR China; c Shenzhen Institute for Technology Innovation, National Institute of Metrology, Shenzhen, 518055, PR China

**Keywords:** *In vitro* cell Experiments, Liquid chromatography-mass spectrometry (LC-MS/MS), Cell density, Drug treatment effect

## Abstract

*In vitro* cell biology study plays a fundamental role in biological and drug development research, but the repeatability and accuracy of cell studies remain to be low. Various uncertainties during the cell culture process could introduce bias into drug research. In this study, we evaluate the potential effects and underlying mechanisms induced by cell number differences in the cell seeding process. Normally, drug experiments are initiated 24 h after cell seeding, and the difference in the cell number at the time of inoculation leads to the difference in cell confluence (cell density) when drug research is conducted. While cell confluence is closely related to intercellular communication, surface protein interaction, cell autocrine as well as paracrine protein expression of cells, it might have a potential impact on the effect of biological studies such as drug treatment. This study used proteomics technology to comprehensively explore the different protein expression patterns between cells with different confluences. Due to the high sensitivity and high throughput of liquid chromatography-mass spectrometry (LC-MS/MS) detection, it was hired to evaluate the protein expression differences of Hep3B cells with 3 different confluences (30%, 50%, and 70%). The differential expressed proteins were analyzed by the Reactome pathway and the Gene Ontology (GO) pathway. Significant differences were identified across three confluences in terms of the number of proteins identified, the protein expression pattern, and the expression level of certain KEGG pathways. We found that those proteins involved in the cell cycle pathway were differently expressed: the higher the cell confluence, the higher these proteins expressed. A cell cycle inhibitor palbociclib was selected to further verify this observation. Palbociclib in the same dose was applied to cells with different confluence, the results indicated that the growth inhibition effect of palbociclib increases along with the increasing trend of cell cycle protein expression. The result indicated that cell density did influence the effect of drug treatment. Furthermore, three other drugs, cisplatin, paclitaxel, and imatinib, were used to treat the three liver cancer cell lines Hep3B, SUN387, and MHCC97, and a similar observation was obtained that drug effect would be different when the cell confluences were different. Therefore, selecting an appropriate number of cells for plating is vitally important at the beginning of a drug study.

## Highlights

• Cell confluence could introduce bias in drug study.
• The expression level of proteins involved in cell cycle pathway increased as cell confluence increase.
• The efficiency of targeted drug might be depended on the expression of its corresponding target protein in cell models.
• The monitoring of cell status by Mass Spectrometry proteomics could facilitate drug validation *in vitro*.

## 1 CLC number: document code: A article IC

Targeted drugs work to target certain proteins that are especially expressed in tumors, and their efficacy depends on the expression of genes and proteins in related pathways [[1], [2], [3]]. When the state of the cell affects the expression of these proteins, the effect of the drug is also affected, thus affecting the accuracy of drug experiments. *In vitro*, cultured tumor cells are the main tools for the studies of tumor diseases. An accurate understanding of cell status by appropriate methods is the first step toward the successful development of tumor drugs. Cell density is one of the key factors that determine the status of the cultured tumor cell which is dynamically changed. *In vitro*, cultured tumor cells could continuously proliferate when nutrition and space are sufficient. For attachment tumor cells, the cell confluence (cell density) would gradually increase as the cells keep proliferation and dividing, therefore the intracellular cell communication would increase, the autocrine paracrine effect become strengthened, and the expression of various proteins both in cell plasma and on cell surface would also change [[4], [5], [6]]. These altered protein expression trends have a potential impact on the subsequent biological and target drug verification experiments.

Proteins constitute the vast majority of drug targets and are the beginning of the drug design and drug use process. Proteins are also the main undertakers of life activities, the direct study of protein is of great significance since it can reveal the essence of life status. However, proteins are highly dynamic and are regulated by various post-translational modifications, subcellular localization, and protein-biomolecular interactions when living cells are proliferating [7]. Traditional two-dimensional electrophoresis can hardly analyze these highly dynamic proteins synchronously, restricted by its loading amount limitation, detection limitation, and lack of specificity and repeatability. As a high throughput method to deeply study multiple proteins and their interaction at the same time, the concept of proteome and proteomics first arose in 1994, afterward, new technology and brilliant research ideas for proteomics were brought out. Mass spectrometry proteome technology can be used to comprehensively study all proteins expressed by a full set of proteins in organisms, tissues, or cells [8]. Liquid chromatography-mass spectrometry (LC-MS/MS) is a powerful tool with the advantages of high throughput and high sensitivity for protein analysis, and LC-MS/MS could analysis quantitatively [9,10]. There are two methods for protein quantification: label-based and label-free. Compared with the introduction of stable isotope-labeled peptides, which introduce the expected mass difference under two or more experimental conditions, label-free proteomics quantifies relative and absolute protein quantities by using the signal intensity and spectral count of peptides [[11], [12], [13], [14], [15]]. Mass spectrometry proteomics is a gradually mature technology to explore drug mechanisms and has been applied to the drug industry for a long time [16]. Using proteomics to study the interrelationship between protein expression and modification in drug therapy can provide important insights for drug development and promote the clinical application of drugs [17,18].

In this study, we aim to use proteomic analysis to reveal the different protein expression patterns when cells were in different confluence. Classical data-dependent property spectrometric mass spectrometry scanning technology was used to detect proteomics, and label-free technology was hired for quantitative high-throughput analysis. It was found that protein expression patterns and their involved Cell Cycle pathways of Hep3B cells were changed along with the cell confluence increase. And the corresponding cell cycle target drug was used to verify the effect of these protein changes.

## 2 Materials and methods

### 2.1 Equipment

Thermo Orbitrap Fusion Lumos high-resolution mass spectrometer, Easy-NLC 1200 NL liquid chromatography system, incubator, high speed centrifuge, ultramicro spectrophotometer (NanoDrop OneC), Multiskan Sky microplate reader were purchased from Thermo Fisher, USA; Electric thermostatic incubator was purchased from Shanghai Yiheng Technology Co., LTD. Concentrator Plus was purchased from Eppendorf Germany; Deionized water was prepared by the laboratory's own Mill-Q pure water system (Millipore, USA).

### 2.2 Cell culture

Hep3B (BNCC, Lot # 337952), MHCC97 (BNCC, Lot # 337741), and SUN387 (NCACC, Lot # SCSP-5046) were cultured in a 5% CO_2_ incubator at 37 °C. The medium consisted of 90% RPMI-I640 (Solarblo, #31800) and 10% fetal bovine serum (EveryGreen, #11011–8611). 10 μM of palbociclib (Selleckchem, S1116) was dissolved in distilled water and diluted with cell medium to a working concentration of 8 μM. Cisplatin (Sigma-Aldrich, RAB7778) was diluted to 6.67 mM in distilled water and diluted to a working concentration of 3 μM in a cell medium. Imatinib (Selleckchem, S2475) and paclitaxel (Aladdin, D2123073) were dissolved in DMSO to an initial concentration of 100 mM. During the experiment, the drug was diluted to 5 μM and 50 nM working concentrations, respectively. The vehicle controls were set by adding equal volumes of solvent (H_2_O or DMSO).

### 2.3 MTT assay

Hep3B, SUN387, and MHCC97 cells were seeded in 96-well plates at a density of 500–4000 cells/well and allowed to attach overnight before drug administration. After palbociclib, cisplatin, imatinib, and paclitaxel were applied to cells, MTT assay (3-(4, 5-dimethyl-1, 3-thiazole-2)-2, 5-dibenzo-2-hydrotetrazole-3-ammonium bromide; Aladdin, #J21061611) was hired to assess cell viability. MTT solution of 5 mg/mL was added to the medium and then incubated at 37 °C for 4 h. Absorbance was measured at OD 490 nm. The growth inhibition rate of cells in each well was calculated as (OD value control -OD value drug)/OD value control × 100%.

### 2.4 LC-MS/MS sample preparation

#### 2.4.1 Cell lysis

When the confluence of cells reached 30%, 50%, and 70%, the cells were digested by adding 0.25% EDTA-trypsin (Solarblo, T1300), centrifuged at 1200 r/min for 5 min, and the cells were precipitated and washed with PBS. After the supernatant was discarded, the cells were lysed with RIPA (Solarblo, #R0020-100) lysate, and the cells were centrifuged at 16000 r/min at 4 °C for 30 min. The supernatant was obtained, and the protein concentration of the supernatant was detected by BCA (Biyuntian, #P0010) method.

### 2.5 Sample preparation

500 μg protein was added into a 30 KD ultrafiltration tube for filter-aided sample preparation (FASP) digestion. After washing the protein with 8 M Urea (UA Sigma-Aldrich, Lot # SLCB4221), the reduction reaction was conducted by adding 100 μl of 20 mM 1,4-Dithiothreitol (DTT, SIGMA, Lot# SLCF2685) to the protein and incubated at 37 °C for 4 h, and then centrifuged for 15 min at 14000 r/min to discard the solution. Next 100 μl of 50 mM 3-Indole acetic acid (IAA, SIGMA, Lot# SLCC6164) was added for alkylation. The mixture was incubated for 30 min at room temperature in the dark and centrifuged for 15 min at 14000 r/min to discard the solution. Afterward, 200 μl of 8 M UA and NH_4_HCO_3_ (Sigma-Aldrich, Lot # BCBV0122) were successfully added three times for washing. Finally, trypsin (Enzyme & Spectrum, P01001) (Enzyme mass: Protein mass = 1: 25) was added, and the protein was incubated at 37 °C for 16 h, peptide samples were collected by centrifuge at 14000 r/min for 15 min, and then 100 μl of ddH_2_O and 200 μl of NH_4_HCO_3_ was added to elute samples, respectively, and centrifuged at 45 °C for hot drying, waiting for loading.

### 2.6 5 data acquisition

Data dependence (DDA) [19] mode was used to collect data from the three samples, and each sample was separated and detected by Easy-nLC 1200 tandem Orbitrap Fusion Lumos. After dilution and dissolution of the sample with 0.1% formic acid, the concentration of peptide was measured by ultramicro spectrophotometer, and the loading volume was 1 μg. The inner diameter of the separation column was 75 μm, the length was 25 cm (reprosil-pur c18-AQ, 1.9 μm; Dr. Maisch), mobile phase: phase A was 0.1 %FA-H_2_O; Phase B is 0.1% FA-80 %ACN; Elution gradients: 0–5 min (4–10% B), 5–48 min (10–22% B), 48–66 min (22–35% B), 66–76 min (35–90% B), 76–78 min (90% B). MS acquisition conditions were set as follows: fragmentation mode was high-energy collision dissociation (HCD), ionization mode was nano-ESI, and positive ion mode was used for scanning. For primary mass spectrometry, the ion scan range is 350–1550 *m*/*z*, the resolution is 120,000, the Automated gain control (AGC) is 40,000, and the maximum ion injection time is 50 ms. For the secondary scan, the resolution was 15,000, the normalized collision energy was 30%, the AGC was 50,000, the maximum ion implantation time was 50 ms, and the mass data were acquired in real-time by XCalibur.

### 2.7 Data analysis

MaxQuant (version 2.0.3.0) software was used to search the original files generated by LC-MS/MS, and Human Uniprot (version 20210902, 20,375 sequences) was used to search the database. The search parameters were set as follows: trypsin was used as a proteolytic enzyme, the maximum missed cleavage site was 2, and the fixed modification was Carbamidomenthylation (C). Variable modifications were set as Acetyl (Protein Nterm), and Oxidation (M); The parent ion Mass tolerance was 20 PPM and the daughter ion Mass tolerance was 4.5 PPM. FDR value (False discovery rates) less than 1%; Other parameters were kept as default values.

“Perseus 2.0.3.0″, a proteome analysis software based on R language, was used for bioinformatics analysis [20,21]. The cell protein expression intensity of Hep3B cells from the MaxQuant result file was used, and the intensity was normalized to the median for statistical analysis. Pathway enrichment was performed using the Metascape online analysis platform [22], which contains database information such as KEGG (Kyoto Encyclopedia of Genes and Genomes), Reactome, and GO (Gene ontology. lysis and processing).

## 3 Results

### 3.1 The difference in cell density leads to the difference in proteome expression

**Fig. 1:**
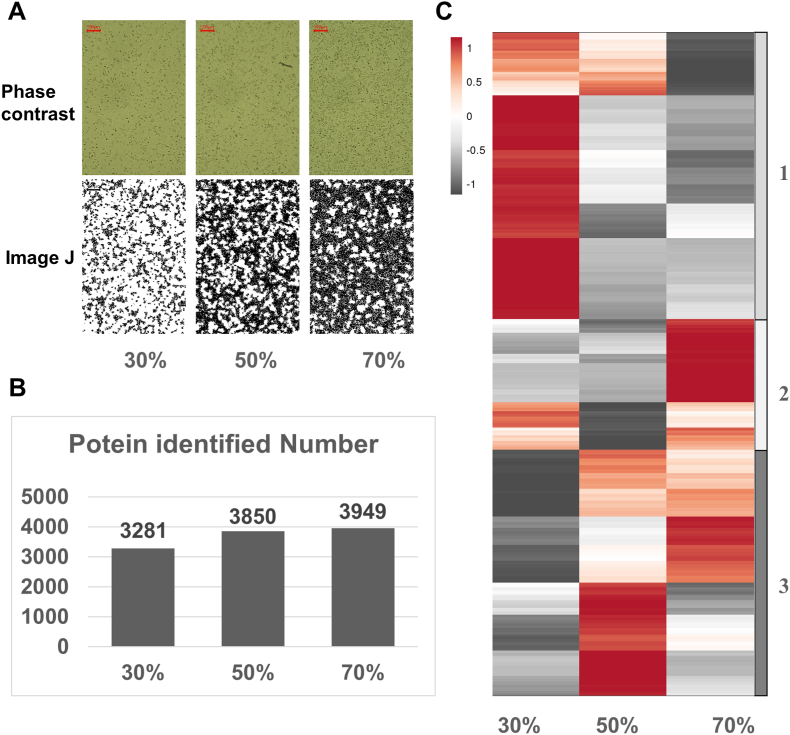
Proteomic profiles of cells with different confluences. (A) Phase contrast (up panel) and Image J processed (low panel) images of cells with 30%, 50%, and 70% confluence. (B) Number of Proteins identified at 30%, 50%, and 70% confluent cells. (C) Heatmap of differentially expressed proteins in cells at 30%, 50%, and 70% confluency.

The difference in proteome expression induced by the difference in cell confluence was shown in Fig. 1. In the experiment, the whole proteome analysis was performed when the Hep3B cell line grew to 30%, 50%, and 70% confluence. As shown in Fig. 1A, the cell culture was terminated when Hep3B cells proliferated to 30%, 50%, and 70% density with Image J software. And the proteins were extracted for mass spectrometry detection. Fig. 1B showed that 3281 proteins were identified when the cell confluence was 30%, which was comparatively less than cells with a confluence of 50% and 70% (3850, and 3949 respectively). This result indicated that a large number of proteins were not yet expressed in Hep3B cells when the confluence reached 30% (low density). At low confluence, the target proteins of some targeted drugs in the experimental cells may not be expressed yet, and the accuracy of target drug validation experiments at this time would be affected. The results of the heat map in Fig. 1C showed that the overall protein expression level of the same cell line would be greatly different when the cell confluence was different. The differently expressed proteins were divided into three groups by whole histone clustering analysis. Proteins in cluster 1 were highly expressed at 30% confluence, and proteins in cluster 2 were significantly highly expressed at 70% confluence. The protein expression pattern in cluster 3 was not as obvious as that in clusters 1 and 2, and the overall expression was high at 50% confluence.

### 3.2 The differential expression of cell cycle proteins at different confluence degrees

**Fig. 2:**
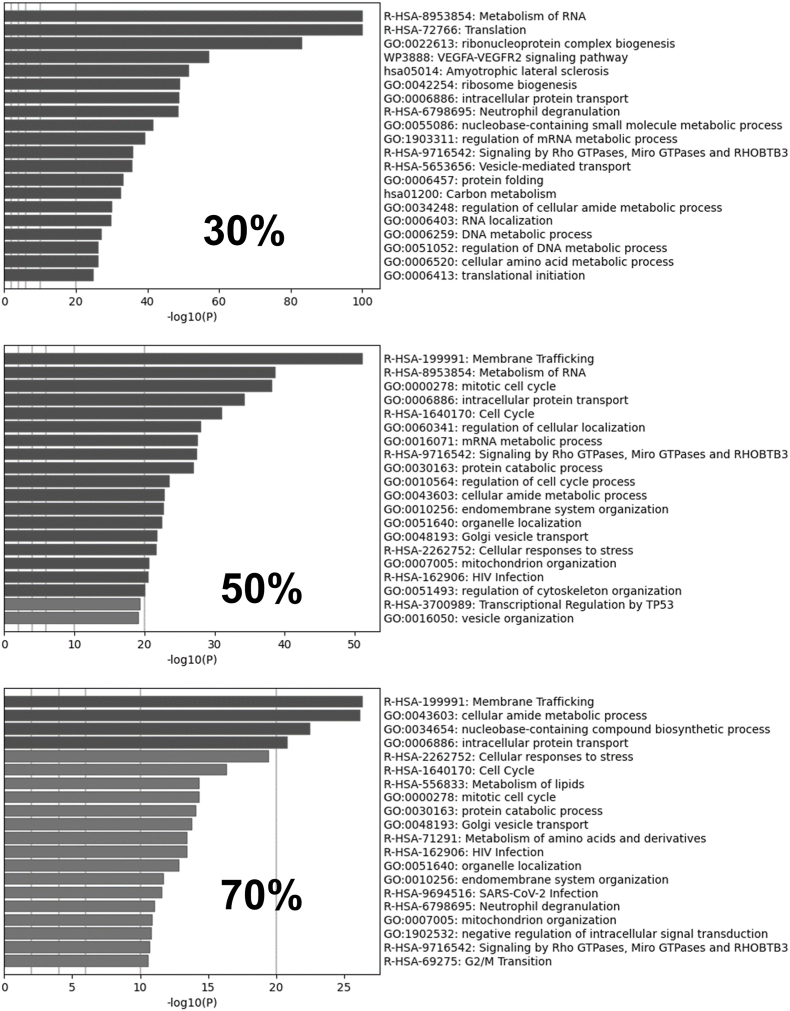
Proteomic profiles of different confluence cells. Enrichment results through Metascape indication proteins from different confluent cells involved in different pathways.

Metascape online platform was hired to enrich the proteins detected from three study groups. According to the pathway enrichment results in Fig. 2, proteins with 30% confluence (cluster 1) of cells were mainly involved in the Metabolism of RNA (R-HSA), translation (R-HSA) from the Reactome database, and ribonucleoprotein complex biogenesis (GO) from Gene ontology database. The proteins highly expressed in 50% confluence （cluster 3）cells were involved in Membrane trafficking, metabolism of RNA, and cell cycle pathway from the Reactome database, as well as mitotic cell cycle and intracellular protein transport (GO). Proteins highly expressed in cells at 70% confluence (cluster 2) were mainly involved in the Reactome signaling pathways including membrane trafficking, cellular responses to stress, and cell cycle, as well as biological process (GO) of intracellular protein transport, nucleobase-containing compound biosynthetic process. The enrichment results suggested that the proteins of the three confluent cells were involved in different stages of cell synthesis. Specifically, cells of 50% and 70% confluence but not 30% confluence were involved in the cell cycle pathway.

### 3.3 The differently expressed cell cycle proteins induced variant cell confluence could introduce bias in cell cycle drug evaluation

**Fig. 3:**
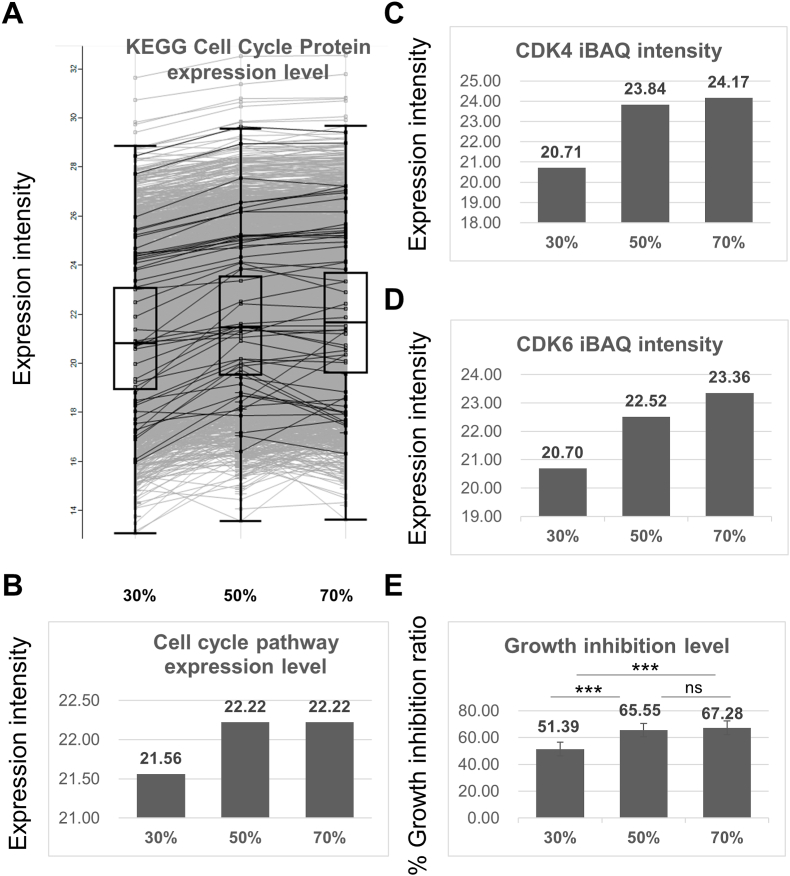
Cell Cycle protein expression level indicated drug response of cell cycle drug. (A) Each Black line indicated one protein that was involved in the cell cycle pathway (KEGG), and the normalized related expression value was retrieved through Perseus software. (B) The overall normalized related expression value of all cell cycle proteins (KEGG). (C) The normalized related expression value of CDK4. (D) The normalized related expression value of CDK6. (E) Growth inhibition of 8 μM Palbociclib treatment for Hep3B cells at 30%, 50%, and 70% confluency.

To further study if the expression of cell cycle proteins would change as the confluence increases, all cell cycle-related proteins expressed in Hep3B cells at the confluence of 30%, 50%, and 70% matching to the KEGG database were retrieved by Perseus (Table 1). And the normalized expression trend of each cell cycle protein (Fig. 3A) and the total cell cycle pathway expression trend (Fig. 3B) were retrieved through Perseus software. The results indicated that the expression of cell cycle proteins in the 30% confluence of Hep3B was comparatively lower than that in the other two groups, and the expression of cell cycle proteins generally increased with the confluence increased. Specifically, the expression of two important cell cycle kinases CDK4 and CDK6 also increased as confluence increased in a similar trend. Whether these differently expressed proteins would affect the drug effect was further tested here. We chose a specific CDK4/6 inhibitor palbocilib [23] to verify if the expression of target proteins would affect the efficiency of the corresponding target drug which are kinases that control cells to enter into the cell cycle [24]. In this study, Hep3B cells with the confluence of 30%, 50%, and 70% were treated with palbociclib of the same concentration. As shown in Fig. 3E, when the cells with the confluence of 30% were treated with palbociclib at the concentration of 8 μM, the inhibition rate of the cells was significantly lower than that of the 50% and 70% groups. And the inhibition rate of 50% was slightly lower than 70%. This changing trend in drug efficiency was the same as the changing trends in the expression of CDK4, CDK6, and the whole cell cycle pathway. In general, for 3 study groups, the growth inhibition rate was closely related to the expression of drug target protein (CDK4 and CDK6) and drug target pathway.

**Table 1:** Cell cycle-related protein expression levels for the three confluences.

Protein IDs	Gene names	iBAQ 30	iBAQ 50	iBAQ 70
O43684	BUB3	24.51	24.74	25.23
O43913	ORC5	18.49	19.17	17.48
O60216	RAD21	20.91	21.06	21.38
O60566	BUB1B	17.75	19.90	20.37
O95067	CCNB2	NaN	18.81	18.04
P06493	CDK1	23.38	24.33	24.78
P11802	CDK4	20.71	23.84	24.17
P12004	PCNA	24.70	26.33	27.24
P14635	CCNB1	20.75	21.50	20.74
P20248	CCNA2	NaN	NaN	17.19
P24941	CDK2	19.99	21.58	20.63
P25205	MCM3	24.23	25.08	25.33
P27348	YWHAQ	25.99	27.55	27.25
P30260	CDC27	15.99	18.27	18.56
P31946	YWHAB	25.42	26.58	27.06
P31947	SFN	25.17	26.19	26.19
P33981	TTK	NaN	18.13	NaN
P33991	MCM4	23.86	25.17	25.42
P33992	MCM5	24.47	25.25	25.23
P33993	MCM7	24.40	25.03	25.36
P42771	CDKN2A	22.51	24.10	22.92
P49736	MCM2	23.81	24.81	24.96
P49841	GSK3B	20.91	21.61	21.89
P50613	CDK7	17.55	18.12	20.36
P51946	CCNH	NaN	19.61	17.53
P53350	PLK1	18.37	20.19	19.57
P61981	YWHAG	25.48	26.56	26.98
P62258	YWHAE	28.46	29.67	29.42
P62877	RBX1	23.10	24.13	23.83
P63104	YWHAZ	27.74	28.97	28.99
P63208	SKP1	24.12	24.93	25.69
P78527	PRKDC	24.27	25.21	25.54
Q00534	CDK6	20.70	22.52	23.36
Q04917	YWHAH	25.25	26.20	26.74
Q09472	EP300	NaN	17.18	17.70
Q13042	CDC16	17.21	20.92	20.11
Q13257	MAD2L1	18.80	22.44	22.25
Q13309	SKP2	NaN	18.45	21.34
Q13416	ORC2	18.06	17.87	17.94
Q13485	SMAD4	18.30	20.03	20.53
Q13547	HDAC1	23.03	23.39	24.13
Q13616	CUL1	19.38	21.53	21.54
Q14566	MCM6	24.38	25.22	25.13
Q14683	SMC1A	19.06	21.34	21.35
Q15796	SMAD2	21.38	21.19	22.43
Q8N3U4	STAG2	16.11	18.71	18.25
Q92769	HDAC2	21.92	23.89	23.36
Q9H1A4	ANAPC1	NaN	19.43	18.86
Q9UBD5	ORC3	17.03	19.74	17.63
Q9UJX2	CDC23	16.91	20.00	17.92
Q9UJX3	ANAPC7	19.25	20.21	20.03
Q9UJX5	ANAPC4	NaN	16.42	18.64
Q9UJX6	ANAPC2	NaN	17.07	16.34
Q9UQE7	SMC3	20.60	21.37	21.21
Q9Y6D9	MAD1L1	17.31	18.71	18.01

**Fig. 4:**
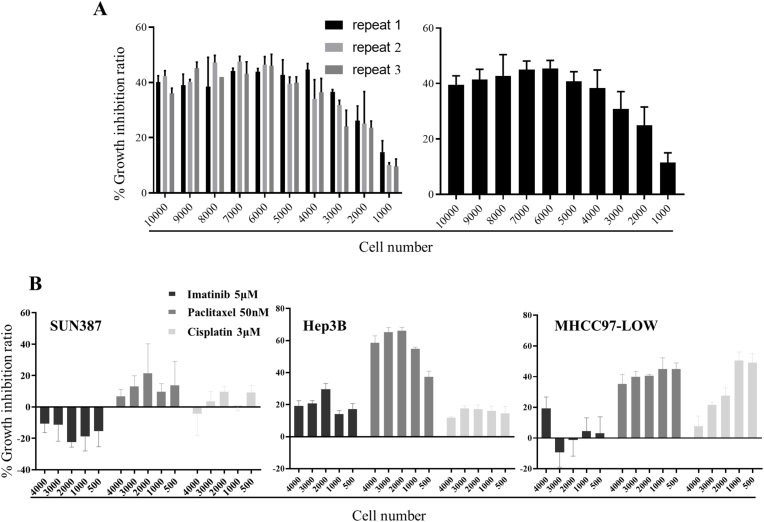
Cell confluence affects the evaluation of drug efficiency. (A) Three repetitions of 8 μM Palbociclib treatment Hep3B cells (Left panel) and pulled data (Right panel). (B)The growth inhibition of 5 μM Imatinib, 50 nM Paclitaxel, and 3 μM Cisplatin on three HCC cell lines of SUN387, Hep3B, and MHCC97-LOW, respectively.

To further verify the relationship between cell confluence and drug efficacy, the cells were refined into 1000, 2000, 3000, 4000, 5000, 6000, 7000, 8000, and 10000 cells per well (96 well plate), and the experiment was repeated for three times (Fig. 3A). These different initiation cell numbers would induce different confluence of the cells when they receive drug treatment. The results indicated that the inhibition rate of palbociclib on Hep3B cells increased firstly and then decreased with the increase of cell confluence. To prove the above inference, three Hepatocellular Carcinoma (HCC) cell lines including Hep3B, SUN387, and MHCC97, were used for verification. Five experimental groups with different cell confluence were set up (starting with 500, 1000, 2000, 3000, and 4000 cells per well). Three drugs, including chemotherapy drugs cisplatin [25], paclitaxel [26], and targeted drugs imatinib [27] were used to treat different experimental groups for three days. Drug inhibition rate results were indicated in Fig. 4, in general, the growth inhibition rate was different when the cell number (cell confluence) became different. These results agreed that tumor cell confluence has an important influence on the test results of tumor drugs.

## 4 Discussion

When using *in vitro* cell models of adherent tumors, increasing cell confluence has a great impact on cell differentiation, cell-matrix interactions, and cell response to drugs [28]. The confluence of cells *in vitro* is not only related to the medium nutrients that each cell could evenly distribute, the autocrine paracrine, and the overall metabolite secretion but also related to the expression and modification of various proteins that contact and communicate between cells, thus affecting the overall state of cells [29].

Malak Bita et al. reported that the differences in cell confluence of human osteosarcoma cell line MG-63 cells would affect the gene transcription levels of alkaline phosphatase (ALPL), RUNT-related transcription factor 2 (RUNX2), and osteonectin (SPARC) [28]. Thereafter affected the performance of cell proliferation, differentiation, and matrix remodeling activities. When Katarina et al. used HeLa cells, they found that there was a significant difference in protein expression between low confluence and high confluence cells [29]. There were significant differences in autophagy-related proteins (P62, LC3B) in different confluence cells, which indicated that autophagy in HeLa cells was aggravated with the increased cell confluence. The differential expression of a series of mTOR pathway proteins related to cell metabolism (mTOR, phosphor-mTOR, S6, pS6) also indicated that cell density has an impact on cell metabolism. Therefore, it is necessary to study those key proteins that correspond to the drug target when using the cell models for experiments. During the cell culture, the expression of key proteins related to the drug target will increase or decrease with the continuous proliferation of cells, or even increase first and then decrease. However, the trends of these changes and their specific effects on experiments have not been thoroughly studied.

Using conventional methods including Western Blot to measure the protein expression of cells is comparatively low efficient, and the number of proteins that could be measured each time is also limited. Understanding the overall state of cells at different confluence requires comprehensive information about proteome expression. The LC-MS/MS has become the mainstream tool for the analysis of proteins from FFPE flux, fresh tissue, body fluids, cells, and other samples as well as small molecules. The tag (Label Free) quantitative proteomic technology could offer relative quantification data without using an isotope label. During mass spectrometry detection, the peptides from different samples would be subjected to different signal strengths, which could be used for the relative quantification of their corresponding proteins. The advantage of label-free technical lies in the fact that proteins do not need to be labeled, therefore the total amount of samples required is less and the consumption is lower. At the same time, the label-free proteome of the cell could facilitate phenotypic analysis and hold great promise for the screening of those drug molecules acting on single or multiple targets that have been validated or not elucidated [30].

In this study, label-free mass spectrometry proteomic data were used to interpret cells at different confluence and predict the drug response. The mass spectrometry proteomic data of hepatocellular carcinoma cell line Hep3B at 30%, 50%, and 70% confluence (density of 30%, 50%, and 70% of the culture surface area) were analyzed. It was found that the total amount of proteins expressed by cells at different confluence was different, and the abundance of different proteins expressed was also different. According to the abundance of protein expression, the proteins expressed by cells were divided into three categories, corresponding to the proteins highly expressed at the confluence of 30%, 50%, and 70%, respectively. Among them, the expression of proteins related to the cell cycle pathway was significantly different among the three groups, which increased with the increase of cell confluence. Hep3B cells were then treated with palbociclib, a drug targeting specific cell cycle initiating proteins CDK4 and CDK6. The results indicated that the drug effect was positively correlated with the expression trend of cell cycle proteins. It is inferred that cell confluence affects the expression of drug target proteins, thereby influencing the results of drug screening. This result is different from the conventional theory that the fewer cells used and the lower the cell density, the more effective the drug response will be obtained since each cell could receive more drug molecules. The experimental results indicated that the drug response was mainly related to the expression abundance of the drug target protein. Therefore, it is speculated that the differential expression of proteins induced by different cell confluence, especially the differential expression of key proteins or protein pathways of drug targets, would largely affect the results of drug validation. Similar results were obtained from other HCC cell lines when using 3 other different drugs. Researchers are encouraged to find the corresponding cell confluence with the highest protein expression for the aiming target drug.

Accurate measurement of cell status, activity, and protein content are of great significance for the accurate use of tumor cell models, which is the basis of life science research. Mass spectrometry proteomic technology is an efficient technique for high-throughput quantitative detection of whole group protein content in cells, which helps accurately grasp the key factors that affect the use of tumor cell models and is of great significance for improving the success rate of drug development, promoting the innovative of de-nove drugs, and realizing precision medicine.

## Authorship

Xinhua Dai and Zhichao Xue brought out the study conception and designed the study, Jiaming Zeng and Bo Meng acquired data. Zhichao Xue and Jiaming Zeng analyzed and interpreted data. Zhichao Xue drafted the article. Yongshu Li, Xiaoyun Gong, and Zhaoyang revised it critically for important intellectual content. Xinhua Dai finally approved the version to be submitted.

## Declaration of competing interest

We declare that we have no financial and personal relationships with other people or organizations that can inappropriately influence our work, there is no professional or other personal interest of any nature or kind in any product, service and/or company that could be construed as influencing the position presented in, or the review of, the manuscript entitled “Mass Spectrometry Proteomics Reveals Cell Density Affects Drug Treatment Efficacy”.

## Data Availability

IPX0004881000 (Original data) (Raw data from Thermo Orbitrap Fusion)
